# Cognitive function and cardiovascular health in the elderly: network analysis based on hypertension, diabetes, cerebrovascular disease, and coronary heart disease

**DOI:** 10.3389/fnagi.2023.1229559

**Published:** 2023-08-04

**Authors:** Yucheng Wang, Huanrui Zhang, Linzi Liu, Zijia Li, Yang Zhou, Jiayan Wei, Yixiao Xu, Yifang Zhou, Yanqing Tang

**Affiliations:** ^1^Department of Psychiatry, The First Hospital of China Medical University, Shenyang, China; ^2^School of Public Health, China Medical University, Shenyang, China; ^3^Department of Geriatrics, The First Hospital of China Medical University, Shenyang, China; ^4^Department of Epidemiology and Biostatistics, Institute of Basic Medical Sciences Chinese Academy of Medical Sciences, Beijing, China; ^5^School of Basic Medicine of Peking Union Medical College, Beijing, China; ^6^Brain Function Research Section, The First Hospital of China Medical University, Shenyang, China

**Keywords:** cognitive domains, aging, vascular factors, network analysis, Ising model

## Abstract

**Introduction:**

Cognitive decline in the elderly population is a growing concern, and vascular factors, such as hypertension, diabetes, cerebrovascular disease, and coronary heart disease, have been associated with cognitive impairments. This study aims to provide deeper insights into the structure of cognitive function networks under these different vascular factors and explore their potential associations with specific cognitive domains.

**Methods:**

Cognitive function was assessed using a modified Chinese version of the mini-mental state examination (MMSE) scale, and intensity centrality and side weights were estimated by network modeling. The network structure of cognitive function was compared across subgroups by including vascular factors as subgroup variables while controlling for comorbidities and confounders.

**Results:**

The results revealed that cerebrovascular disease and coronary heart disease had a more significant impact on cognitive function. Cerebrovascular disease was associated with weaker centrality in memory and spatial orientation, and a sparser cognitive network structure. Coronary heart disease was associated with weaker centrality in memory, repetition, executive function, recall, attention, and calculation, as well as a sparser cognitive network structure. The NCT analyses further highlighted significant differences between the cerebrovascular disease and coronary heart disease groups compared to controls in terms of overall network structure and connection strength.

**Conclusion:**

Our findings suggest that specific cognitive domains may be more vulnerable to impairments in patients with cerebrovascular disease and coronary heart disease. These insights could be used to improve the accuracy and sensitivity of cognitive screening in these patient populations, inform personalized cognitive intervention strategies, and provide a better understanding of the potential mechanisms underlying cognitive decline in patients with vascular diseases.

## Highlights

- First study to use network analysis to examine the relationship between cognitive function and cardiovascular health.
- Cerebrovascular disease group had weaker centrality in memory and spatial orientation.
- Coronary heart disease group had weaker centrality in temporal orientation, immediate recall and delayed recall.
- Study identified potential associations between the four disorders and cognitive impairments.

## 1. Introduction

Dementia is a progressive neurocognitive disorder characterized by insidious cognitive and functional decline until death. Mild cognitive impairment (MCI) typically precedes dementia and is characterized by impairment in one or more cognitive domains (World Health Organization [WHO], 2019; GBD 2019 Dementia Forecasting Collaborators, 2022). With the acceleration of population aging, cognitive disorders, including dementia and MCI, have become a significant public health issue, attracting increasing attention from healthcare providers, researchers, and policymakers (Wu et al., 2017). Cognitive decline is often accompanied by neuropsychiatric symptoms and decreased ability to complete daily living activities, negatively impacting the quality of life for patients and caregivers and imposing a heavy burden on their families and society as a whole (Sun et al., 2018). According to estimates by the International Alzheimer’s Association, there are approximately 50 million people with dementia worldwide, a number that is expected to double by 2050, with two-thirds living in low- and middle-income countries (Alzheimer’s Disease International [ADI], 2018). In China, approximately 15 million people aged 60 and older suffer from dementia, with an estimated overall MCI prevalence of 15.5% (Baumgart et al., 2015). The neuropathological changes associated with cognitive decline begin to progress long before clinical manifestation and mostly occur in older age, providing ample time for the implementation of preventive strategies to effectively delay age-related cognitive decline and dementia (Feldman et al., 2014). As there are currently no effective treatments, reducing the risk of cognitive decline and modulating modifiable risk factors to delay disease onset have become important components of prevention strategies (Scheltens et al., 2021).

Numerous risk factors contribute to cognitive decline with varying mechanisms and scopes of damage, including specific neurobiological differences (Bellou et al., 2017; Zhang et al., 2022). Hypertension, diabetes, cerebrovascular disease, and coronary artery disease, common chronic conditions in elderly individuals, are considered to be closely related to cognitive decline (Kivimäki et al., 2019; Ou et al., 2020; Yang et al., 2020; Andrews et al., 2021; Feter et al., 2022). These four vascular factors can be easily identified and subjected to intervention, making them suitable for preventing the development of cognitive impairment and dementia. Although vascular factors share similar pathological mechanisms, including causing damage to small blood vessels, some phenomenological studies have found that the extent and range of cognitive impairment vary among populations with different vascular factors (Skoog et al., 1996; Ray et al., 2016; Xie et al., 2019). With the deepening of pathophysiological research in recent years, researchers have discovered that different vascular factors have varying mechanisms of damaging cognitive function. They produce different inflammatory factors and endogenous hormones, which bind to their corresponding receptors, leading to impaired synaptic, metabolic, and immune responses, and cause differences in brain structure and neural connections (Skoog et al., 1996; Langbaum et al., 2012; Love and Miners, 2016; Niu et al., 2022). For instance, hypertension may impact cognitive function by influencing cerebral blood flow and causing cerebral microvascular damage (Ungvari et al., 2021), while diabetes primarily results in the accumulation of abnormally folded amyloid-beta peptide (Aβ) and tau protein in amyloid plaques and neurofibrillary tangles, as well as various forms of vascular injury (Moran et al., 2015; Xue et al., 2019). Cerebrovascular disease may disrupt brain tissue structure and function through localized or global cerebral ischemia and induce brain infarction or hemorrhage (Gorelick et al., 2011), whereas coronary artery disease might affect brain blood flow and oxygen supply by impacting cardiac pumping function and causing microemboli (Wolters et al., 2018). The specific range of cognitive impairment caused by different risk factors requires further elucidation to develop personalized intervention measures.

Network analysis has been widely applied in recent years to the research and description of psychological traits and psychiatric symptoms (van Borkulo et al., 2014; Borsboom, 2017). It directly utilizes observable variables, such as attitudes, feelings, and behaviors, as nodes and employs regularization techniques to establish partial correlation networks between these variables (Epskamp et al., 2012). This approach supplements traditional factor analysis and latent variable research methods, providing novel insights for better understanding human psychological phenomena and exploring the structure of psychiatric disorders (Epskamp and Fried, 2018). When cognitive conditions are viewed from a network theory perspective, dimensions and domains interact directly and continuously (van Loo et al., 2018). The presence of different risk factors may affect the pathway and intensity of the connections between these symptoms, potentially leading to individual differences in developmental obstacles.

To date, no studies have used network analysis to explore the potential relationship between cognitive function and cardiovascular health in older adults. In this study, we present cognitive ability scales in a network format to display the characteristics of cognitive function network structure in older adults more clearly. By using vascular factors as grouping variables, we compared the cognitive function network structures of different subgroups and investigated the potential impact of vascular factors on cognitive abilities. Simultaneously, we established graphical models to identify core nodes and modules in cognitive function networks of the various vascular factors, which may serve as key elements for future disease identification and intervention. This approach provides guidance for developing personalized intervention measures tailored to different risk factors.

## 2. Materials and methods

### 2.1. Participants

This cross-sectional survey was conducted from September 2018 to June 2022 using a convenience snowball sampling method. The study population consisted of older adults residing in communities, nursing homes, townships, and hospitals in Liaoning Province, China. Selection criteria included the following: (1) individuals aged 60 years and older; (2) individuals residing in the survey area for at least 6 months; (3) individuals without moderate to severe visual or hearing impairment, motor dysfunction, language barriers, or other conditions that may affect cognitive function measurement; (4) individuals without a clinical diagnosis of dementia or severe mental illness; (5) individuals currently in a stable physical condition without medication non-compliance; and (6) participants who voluntarily participated in the study and signed informed consent forms. Individuals with severe diseases, terminal illnesses, or complete bedridden disability were excluded. The study protocol was approved by the Human Ethics Committee of China Medical University.

### 2.2. Procedure

Upon meeting the study’s inclusion criteria, being informed of the research purpose, and signing the informed consent form, participants underwent clinical interviews conducted by trained researchers. Relevant demographic information (age, sex, education level, hypertension, diabetes, cerebrovascular disease, coronary heart disease, and history of psychological trauma) was collected, and cognitive function assessment tests were completed.

### 2.3. Cognition assessment

The mini-mental state examination (MMSE) is primarily utilized for screening dementia patients, assessing the severity of cognitive impairment, and monitoring disease progression (Tombaugh and McIntyre, 1992). Due to its ease of use and short administration time (5–10 min), the MMSE has been widely adopted both domestically and internationally. In this study, we used the Chinese mini-mental status (CMMS) (Zhang et al., 1990), a Chinese version of the MMSE revised by Zhang Mingyuan, to assess cognitive function. Its validity and reliability have been proven previously (Wu et al., 2022). The CMMS is a fundamental tool for evaluating overall cognitive function, with a maximum achievable score of 30 points. It contains five subsets, orientation, registration, attention and calculation, recall, and language and visual construction, with a total of thirty items, each rated on a scale of 0 to 1.

### 2.4. Data analysis

We used EpiData 3.1 software to input information from paper questionnaires. Data entry and validation were independently completed by two researchers, eliminating any inconsistencies or logical errors in the basic information. After ensuring accuracy, a database was established. We monitored missing data patterns in the collected data and utilized R 4.1.3 software (R Core Team, 2022) and the mice package (van Buuren and Groothuis-Oudshoorn, 2011) for data computation, missing data imputation, and model validation. For values meeting the conditions for multiple imputation, the data were imputed using the software package.

#### 2.4.1. Network model construction

The construction of the network model was performed with R 4.1.3 software (R Core Team, 2022). Networks consist of “nodes” and “edges,” with each symptom considered a node and the association between two symptoms treated as an edge (Borsboom and Cramer, 2013; van Borkulo et al., 2015). Blue and green edges represent positive correlations and red edges represent negative correlations between symptoms, and edge thickness indicates the strength of their association. The magnitude of an edge weight reflects the strength of the association, with higher absolute values indicating stronger associations and lower absolute values indicating weaker associations. Nodes with stronger connections to other nodes are closer to the center of the network, while nodes with fewer connections are distributed around the network’s periphery. Each color node in the network represents a subset (e.g., different cognitive domains, cardiovascular disease prevalence, covariates).

Since the data in this study did not follow a normal distribution, we employed the “IsingFit” package (van Borkulo et al., 2014) in R 4.1.3, using an Ising model based on binary data to construct a computationally efficient model for estimating network structure. To improve the sensitivity of the network model in detecting changes in cognitive function, we encoded the scores of the 30 MMSE items as binary data, with the minimum sample size for each network estimated to be ten times the number of nodes (van der Ploeg et al., 2014). We then applied the graphical least absolute shrinkage and selection operator (GLASSO) to introduce a penalty factor, removing relatively weak connections in the network to obtain a more stable and easily interpretable regularized sparse network. The statistical analysis and visualization features of these networks were implemented using the R packages “qgraph” (Epskamp et al., 2014) and “glasso” (Friedman et al., 2014).

#### 2.4.2. Network estimation and centrality measurements

We estimated network models ranging from simple to complex. In the network models, nodes represent variables, and edges between nodes represent conditional dependencies, which can be understood as partial correlations. Centrality parameters for each node can be assessed as strength, closeness, and betweenness (Bringmann et al., 2013). Since closeness and betweenness may be unreliable in the cognitive network model presented in this study (Bringmann et al., 2019), we primarily focused on the effects of strength centrality. Strength centrality is reported as standardized z scores, a high centrality Z score for a cognitive domain indicates that it plays a more central role in the network, with stronger connections to other nodes and potentially greater impact on cognitive function.

Before interpreting centrality estimates, we assessed network stability using a case deletion bootstrap approach. We removed varying proportions of samples, ranging from 10 to 75%, and estimated the network model using only the remaining data. By calculating the correlation between bootstrap measures for the subset models and the original subset models, we evaluated the robustness of the estimated parameters, which should theoretically be higher than 0.25 (Armour et al., 2017).

#### 2.4.3. Covariates and network comparisons

We explored the potential effects of four disease factors on cognitive abilities by comparing the cognitive function network structures of subgroups using these factors as grouping variables. Specifically, we divided the population into eight subsets within four subgroups: with and without diabetes, with and without hypertension, with and without cerebrovascular disease, and with and without coronary heart disease. Based on previous research (Harada et al., 2013; Baumgart et al., 2015; Maccora et al., 2020; Levine et al., 2021), we selected sex, age, and educational level as covariates. For each subgroup, we included the presence of the other three diseases, sex, age, and educational level as covariates in the network to control for comorbidities and other confounding factors related to cognition that may affect the cognitive network structure. The network comparison test (NCT) was used to compare the overall network structure and the global strength of connections of the two subset cognitive network models within each subgroup (van Borkulo et al., 2022) to explore the potential impact of the presence or absence of the cardiovascular health-related factors under investigation on the cognitive network structure. “Overall network structure” refers to the assumption that the entire network (i.e., the specific pattern of edges connecting nodes) is the same between groups. “Global strength of connections” refers to the assumption that the total absolute sum of all edges is the same across the network.

In all analyses, results with *p* < 0.05 were considered statistically significant. The code for network analysis can be found at https://osf.io/vh825/?view_only=e18be553d28047788e5dfbc9303a2817. We also provide the model outputs to make the analysis reproducible.

## 3. Results

### 3.1. Sample characteristics

A total of 2,225 valid questionnaires were collected. The study included 1,152 men (51.8%) and 1,073 women (48.2%). The average age was 72.44 ± 9.63 years. Regarding education level, 400 participants (18.0%) had a college degree or higher, 991 (44.5%) had a high school education, 781 (35.1%) had a primary school education, and 53 (2.4%) were illiterate. There were 921 individuals (41.3%) with hypertension, 381 (17.1%) with diabetes, 732 (32.8%) with cerebrovascular disease, and 484 (21.7%) with coronary heart disease. The average MMSE score for the study population was 26.21 ± 3.16. Table 1 displayed the recoded MMSE items.

**TABLE 1:** Recoded mini-mental state examination items and frequencies (%).

Community		Label	Item description	Failure to answer questions or incomplete answers	Answer the question correctly
I. Orientation (10 points)	Temporal orientation	OT1	What is the day?	312 (14%)	1,913 (86%)
		OT2	What is the date?	328 (14.7%)	1,897 (85.3%)
		OT3	What is the month?	89 (4%)	2,136 (96%)
		OT4	What is the season?	49 (2.2%)	2,176 (97.8%)
		OT5	What is the year?	101 (4.5%)	2,124 (95.5%)
	Spatial orientation	OS1	Where are we now? Province?	13 (0.6%)	2,212 (99.4%)
		OS2	What district do you live in?	31 (1.4%)	2,194 (98.6%)
		OS3	What street do you live in?	118 (5.3%)	2,107 (94.7%)
		OS4	Where is this place?	63 (2.8%)	2,162 (97.2%)
		OS5	Where are we now? Floor?	77 (3.5%)	2,148 (96.5%)
II. Registration (3 points)	Immediate recall	R1	Repeat the words: ball	97 (4.4%)	2,128 (95.6%)
		R2	Repeat the words: National Flag	103 (4.6%)	2,122 (95.4%)
		R3	Repeat the words: tree	154 (6.9%)	2,071 (93.1%)
III. Attention and calculation (5 points)	Attention and calculation	A1	100 − 7 (93)	72 (3.2%)	2,153 (96.8%)
		A2	−7 (86)	509 (22.9%)	1,716 (77.1%)
		A3	−7 (79)	605 (27.2%)	1,620 (72.8%)
		A4	−7 (72)	678 (30.5%)	1,547 (69.5%)
		A5	−7 (65)	830 (37.3%)	1,395 (62.7%)
IV. Recall (3 points)	Delayed recall	D1	Recall: ball	535 (24.0%)	1,690 (76.0%)
		D2	Recall: national flag	655 (29.4%)	1,570 (70.6%)
		D3	Recall: tree	798 (35.9%)	1,427 (64.1%)
V. Language and visual construction (9 points)	Naming	LN1	Name shown object: watch	24 (1.1%)	2,201 (98.9%)
		LN2	Name shown object: pencil	28 (1.3%)	2,197 (98.7%)
	Repetition	LP1	Repeat the phrase	309 (13.9%)	1,916 (86.1%)
	Executive function	LE1	Follow oral instructions.1	142 (6.4%)	2,083 (93.6%)
		LE2	Follow oral instructions.2	108 (4.9%)	2,117 (95.1%)
		LE3	Follow oral instructions.3	148 (6.7%)	2,077 (93.3%)
	Reading	LR1	Follow written instruction	242 (10.9%)	1,983 (89.1%)
	Expression	LEx1	Make up and write a sentence	604 (27.1%)	1,621 (72.9%)
	Drawing	LD1	Copy a picture (of two figures)	659 (29.6%)	1,566 (70.4%)

### 3.2. MMSE total score model

**FIGURE 1:**
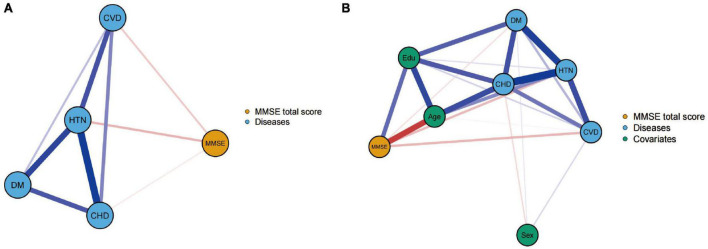
Networks showing the mini-mental state examination (MMSE) total score model **(A)** and the model after controlling for covariates **(B)**. The thicknesses of lines represent the strength of the correlation. Blue lines represent positive correlations, whereas red lines represent negative correlations.

To investigate the network relationships between the MMSE total score and cerebrovascular disease, coronary heart disease, hypertension, and diabetes in the elderly population, two network model structures were constructed, as shown in Figure 1. Figure 1A displayed a negative correlation between the MMSE total score and the prevalence of the four vascular diseases in the elderly population. Additionally, there was a positive association among the four diseases. Figure 1B is based on Figure 1A with the additional three covariates of sex, age, and educational level. After adjusting for these covariates, the association patterns and strengths between the MMSE total score and the four diseases changed. This suggests that when analyzing the relationship between cognitive function and cardiovascular factors, potential confounders such as age, sex, and educational level need to be considered. These factors may have an impact on cognitive function or interact with the four disease factors.

### 3.3. MMSE individual score items network model

**FIGURE 2:**
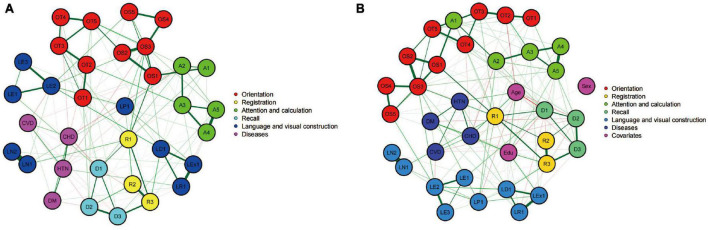
Networks showing the mini-mental state examination (MMSE) individual score model **(A)** and the model after controlling for covariates **(B)**. Circles indicate nodes (MMSE item) and lines indicate edges (association between two symptoms). The thicknesses of lines represent the strength of the correlation. Nodes with the same color belong to the same community. Green lines represent positive correlations, whereas red lines represent negative correlations.

Next, two network models, as shown in Figure 2, were constructed to evaluate the relationship between the 30 MMSE items individually and the prevalence of the four diseases in the entire population. Figure 2A presents a network model without considering sex, age, and educational level. This model showed extensive connections among different cognitive domains and within individual cognitive domains, as well as connections between the four diseases and various cognitive domains. In this model, the strength centrality z scores for hypertension, diabetes, cerebrovascular disease, and coronary heart disease were −1.696, −0.478, −1.679, and −0.903, respectively, indicating their relative importance in the network.

Figure 2B incorporated sex, age, and educational level as covariates into the cognitive network model. This led to a change and reorganization of the cognitive function network structure. After adding these covariates, the nodes representing the four diseases occupied more central positions in the network, and the connections with various cognitive domains were strengthened. In this adjusted model, the strength centrality z scores for hypertension, diabetes, cerebrovascular disease, and coronary heart disease were −1.533, −0.403, −1.152, and −0.355, respectively. After considering the three covariates of sex, age, and educational level, the importance of the four diseases in the cognitive function network increased. This implies that the impact of the four vascular factors on the cognitive function structure may be modulated by age, sex, and educational level. Subsequent network model construction and comparison will adjust for the influence of these three confounding factors.

### 3.4. Comparison of network models divided by vascular factors

Next, we divided the population into eight subsets within four subgroups according to the presence or absence of hypertension, diabetes, cerebrovascular disease, and coronary heart disease.

#### 3.4.1. Hypertension group

**FIGURE 3:**
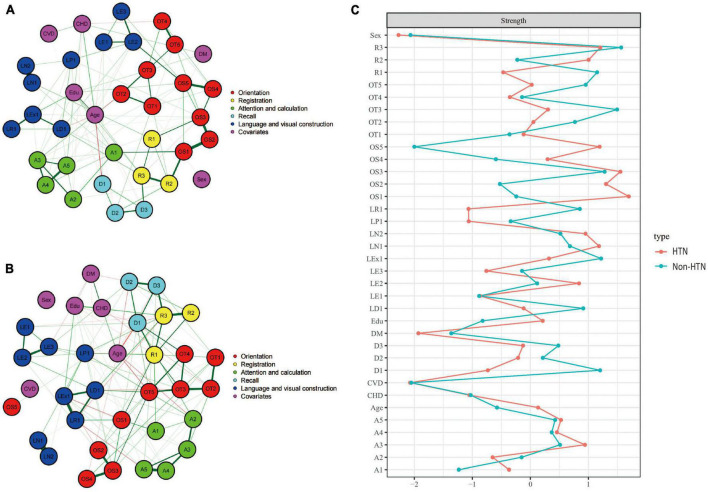
Cognitive network structures in participants with and without hypertension, and centrality indices of each node. **(A)** Shows the cognitive network structure for participants with hypertension, and **(B)** presents the structure for those without hypertension. **(C)** Displays the centrality indices (Z-scores) of each node in the network analysis. Nodes with the same color belong to the same community. Edges indicate the strength and direction of associations between nodes: green lines represent positive correlations, and red lines represent negative correlations. The thickness of the edges corresponds to the magnitude of the correlations, with thicker lines indicating stronger associations. The centrality Z-score reflects the importance of a node in the network; the higher the centrality, the greater its influence within the network.

Figures 3A, B showed the cognitive network structures of the two groups of people with hypertension (*n* = 921) and without hypertension (*n* = 1,304), respectively. Figure 3C displayed the centrality indices of each node in the network analysis. In the group with hypertension, the three items in spatial orientation were the three nodes with the highest centrality (OS1, *z* = 1.698; OS3, *z* = 1.553; OS2, *z* = 1.304). In the group without hypertension, the nodes with the highest centrality were memory (R3, *z* = 1.566), time orientation (OT3, *z* = 1.496), and spatial orientation (OS3, *z* = 1.280).

In the hypertension group, the internal connections of cognitive functions were mostly positive, with 446 positive connections and a sum of non-standardized partial correlation coefficients of 231.48. The most heavily weighted edges occurred in the naming ability module (LN1–LN2, edge weight = 4.113), followed by the spatial orientation module (OS1–OS2, edge weight = 2.816; OS2–OS3, edge weight = 2.210). In the group without hypertension, there were 425 positive connections, with a sum of partial correlation coefficients of 300.152. The most heavily weighted edges occurred in the naming ability module, followed by the attention and calculation module (A4–A5, edge weight = 2.816) and the connection between reading ability and writing ability modules (LR1-LEx1, edge weight = 2.703). Edge weights with 95% confidence intervals are available in the Supplementary information.^1^

The NCT results showed that there were no significant differences in the overall network structure between the two groups (hypertension group vs. non-hypertension group: *M* = 2.783, *p* = 0.415). In terms of global connectivity strength, there was also no significant difference between the two groups (hypertension group vs. non-hypertension group: *S* = 10.86, *p* = 0.275).

#### 3.4.2. Diabetes group

**FIGURE 4:**
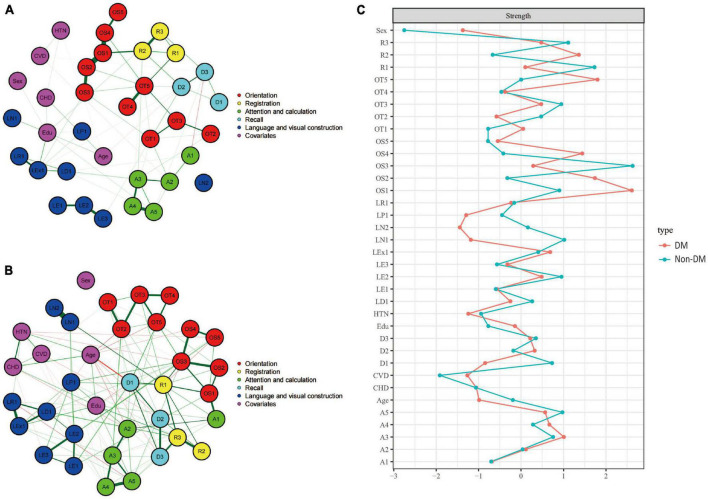
Cognitive network structures in participants with and without diabetes, and centrality indices of each node. **(A)** Shows the cognitive network structure for participants with diabetes, and **(B)** presents the structure for those without diabetes. **(C)** Displays the centrality indices (Z-scores) of each node in the network analysis. Nodes with the same color belong to the same community. Edges indicate the strength and direction of associations between nodes: green lines represent positive correlations, and red lines represent negative correlations. The thickness of the edges corresponds to the magnitude of the correlations, with thicker lines indicating stronger associations. The centrality Z-score reflects the importance of a node in the network; the higher the centrality, the greater its influence within the network.

Figures 4A, B showed the cognitive network structures of the two groups of people with diabetes (*n* = 381) and without diabetes (*n* = 1,844), respectively. Figure 4C displayed the centrality indices of each node in the network analysis. In the group with diabetes, the node with the highest centrality was OS1 (*z* = 2.608), followed by OT5 (*z* = 1.802) and OS2 (*z* = 1.738). In the group without diabetes, the node with the highest centrality was OS3 (*z* = 2.629), followed by R1 (*z* = 1.731) and R2 (*z* = 1.112).

In the diabetes group, there were 389 positive connections, with a sum of partial correlation coefficients of 379.84. The most heavily weighted edges occurred between the time orientation module and the memory module (OT5-R1, edge weight = 4.122), followed by within the time orientation module (OT4–OT5, edge weight = 3.861) and the executive function module (LE2–LE3, edge weight = 3.018). In the group without diabetes, there were 442 positive connections, with a sum of partial correlation coefficients of 291.04. The most heavily weighted edges occurred in the naming ability module (LN1–LN2, edge weight = 4.847), followed by the attention and calculation module (A4–A5, edge weight = 2.575) and the connection between the reading ability module and the writing ability module (LR1-LEx1, edge weight = 2.453). Edge weights with 95% confidence intervals can be found in the Supplementary information.

The NCT results showed that there were no significant differences in the overall network structure between the two groups (diabetes group vs. non-diabetes group: *M* = 4.730, *p* = 0.267). In terms of global connectivity strength, there was also no significant difference between the two groups (diabetes group vs. non-diabetes group: *S* = 32.24, *p* = 0.467).

#### 3.4.3. Cerebrovascular disease group

**FIGURE 5:**
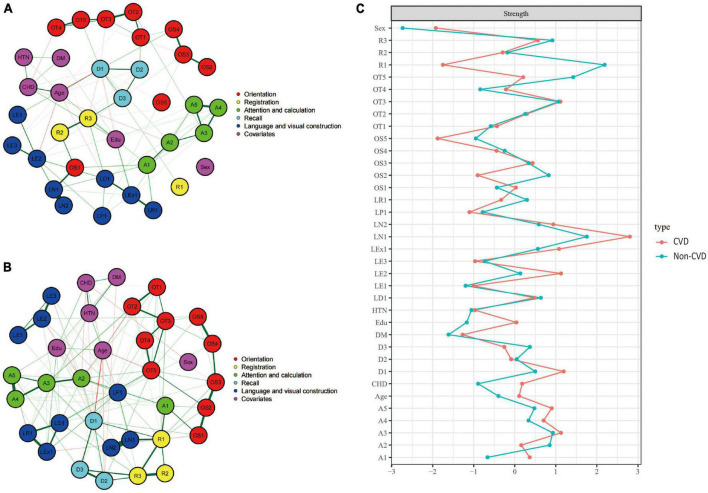
Cognitive network structures in participants with and without cerebrovascular disease, and centrality indices of each node. **(A)** Shows the cognitive network structure for participants with cerebrovascular disease, and **(B)** presents the structure for those without cerebrovascular disease. **(C)** Displays the centrality indices (Z-scores) of each node in the network analysis. Nodes with the same color belong to the same community. Edges indicate the strength and direction of associations between nodes: green lines represent positive correlations, and red lines represent negative correlations. The thickness of the edges corresponds to the magnitude of the correlations, with thicker lines indicating stronger associations. The centrality Z-score reflects the importance of a node in the network; the higher the centrality, the greater its influence within the network.

Figures 5A, B showed the cognitive network structures of the two groups of people with cerebrovascular disease (*n* = 732) and without cerebrovascular disease (*n* = 1,493), respectively. Figure 5C displayed the centrality indices of each node in the network analysis. In the group with cerebrovascular disease, the node with the highest centrality was LN1 (*z* = 2.804), followed by D1 (*z* = 1.192) and A3 (*z* = 1.126). In the group without cerebrovascular disease, the node with the highest centrality was R1 (*z* = 2.189), followed by LN1 (*z* = 1.755) and OT5 (*z* = 1.423).

In the cerebrovascular disease group, there were 419 positive connections, with a sum of partial correlation coefficients of 232.07. The most heavily weighted edges occurred in the naming ability module (LN1–LN2, edge weight = 2.09), followed by the attention and calculation module (A4–A5, edge weight = 2.082) and the time orientation module (OT2–OT3, edge weight = 2.009). In the group without cerebrovascular disease, there were 432 positive connections, with a sum of partial correlation coefficients of 292.15. The most heavily weighted edges occurred in the naming ability module (LN1–LN2, edge weight = 4.917), followed by the attention and calculation module (A4–A5, edge weight = 2.752) and the connection between the reading ability module and the writing ability module (LR1-LEx1, edge weight = 2.549). Edge weights with 95% confidence intervals can be found in Supplementary information. The NCT results showed that there were no significant differences in the overall network structure between the two groups (cerebrovascular disease group vs. non-cerebrovascular disease group: *M* = 2.178, *p* = 0.835). However, there was a significant difference in global connectivity strength between the two groups (cerebrovascular disease group vs. non-cerebrovascular disease group: *S* = 27.218, *p* = 0.045).

#### 3.4.4. Coronary heart disease group

**FIGURE 6:**
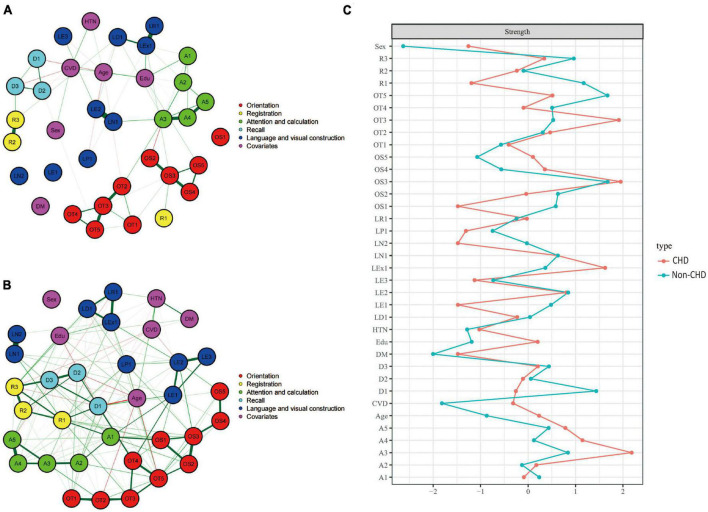
Cognitive network structures in participants with and without coronary heart disease, and centrality indices of each node. **(A)** Shows the cognitive network structure for participants with coronary heart disease, and **(B)** presents the structure for those without coronary heart disease. **(C)** Displays the centrality indices (Z-scores) of each node in the network analysis. Nodes with the same color belong to the same community. Edges indicate the strength and direction of associations between nodes: green lines represent positive correlations, and red lines represent negative correlations. The thickness of the edges corresponds to the magnitude of the correlations, with thicker lines indicating stronger associations. The centrality Z-score reflects the importance of a node in the network; the higher the centrality, the greater its influence within the network.

Figures 6A, B showed the cognitive network structures of the two groups of people with coronary heart disease (*n* = 484) and without coronary heart disease (*n* = 1,741), respectively. Figure 6C displayed the centrality indices of each node in the network analysis. In the group with coronary heart disease, the node with the highest centrality was A3 (*z* = 2.183), followed by OS3 (*z* = 1.952) and OT3 (*z* = 1.914). In the group without coronary heart disease, the node with the highest centrality was OS3 (*z* = 1.678), followed by OT5 (*z* = 1.672) and D1 (*z* = 1.438).

In the coronary heart disease group, there were 405 positive connections, with a sum of partial correlation coefficients of 213.07. The most heavily weighted edges occurred in the naming ability module (LN1–LN2, edge weight = 2.09), followed by the attention and calculation module (A4–A5, edge weight = 2.082) and the time orientation module (OT2–OT3, edge weight = 2.009). In the group without coronary heart disease, there were 421 positive connections, with a sum of partial correlation coefficients of 299.39. The most heavily weighted edges occurred in the naming ability module (LN1–LN2, edge weight = 4.731), followed by the attention and calculation module (A4–A5, edge weight = 2.753) and the connection between the reading ability module and the writing ability module (LR1-LEx1, edge weight = 2.127). Edge weights with 95% confidence intervals can be found in Supplementary information.

The NCT results showed that there were significant differences in the overall network structure between the two groups (coronary heart disease group vs. non-coronary heart disease group: *M* = 4.608, *p* = 0.05). Furthermore, there was a significant difference in global connectivity strength between the two groups (coronary heart disease group vs. non-coronary heart disease group: *S* = 45.386, *p* < 0.001).

## 4. Discussion

To the best of our knowledge, this is the first study to employ network analysis to explore the association between cognitive functioning and cardiovascular health. We presented the structure of the MMSE scale in the form of a network. By dividing the population into four subgroups according to the presence or absence of hypertension, diabetes, cerebrovascular disease, and coronary heart disease and controlling for potential confounding factors, we compared the differences between cognitive network structures to examine the potential impact of these risk factors on cognitive function. Furthermore, by identifying the core nodes of the cognitive networks in each subgroup, we highlight their importance for future disease identification and intervention.

In the first part of this study, we employed network models to estimate the relationship between the total MMSE scores of elderly individuals and cerebrovascular disease, coronary heart disease, hypertension, and diabetes. The results showed a negative correlation between cognitive functioning scores and the prevalence of these four vascular-related diseases, indicating that cognitive functioning might be impaired as these diseases develop and progress. The interrelatedness of the disease prevalence suggests that they may share some similar pathological mechanisms and risk factors (Iadecola, 2010; Gorelick et al., 2011).

In the second part of this study, we assessed the network models for the 30 items of the MMSE and the prevalence of the four diseases for the entire population. The results revealed that the prevalence of the four diseases was associated with network nodes across various cognitive domains, indicating that the presence of these diseases has a certain degree of impact on individual cognitive domains as well as overall cognitive functioning. This finding aligns with the studies by Manschot et al. (2006) and Debette et al. (2010), who found that vascular changes might affect multiple cognitive domains, such as memory, executive function, and attention. Vascular factors may influence cognitive functioning through various pathways, such as ischemia, hypoxia, neuroinflammation, and alterations in cerebrovascular reactivity (Kivipelto et al., 2001; Gorelick et al., 2011).

In the third part of this study, we constructed eight network models within the four disease subgroups to explore the potential impact of these diseases on cognitive functioning. We found that, patients in the hypertension group showed reduced centrality in time orientation, delayed recall, repetition, and reading abilities. However, a compensatory increase in the centrality of spatial orientation was observed. These results provide insights into the differential impact of hypertension on specific cognitive domains. Our findings are consistent with some previous studies that identified an association between hypertension and cognitive decline. For example, a study by Elias et al. (2012) and Sun et al. (2020) suggested that hypertension is related to declines in several cognitive domains, including memory, attention, and executive function. Similarly, Iadecola and colleagues (Iadecola et al., 2016) found that hypertension could lead to cognitive impairments by altering cerebral blood flow and inducing microvascular damage. The compensatory increase in spatial orientation centrality observed in our study is an intriguing finding, suggesting that hypertensive patients may rely more on their spatial orientation abilities to counteract declines in other cognitive functions. This phenomenon has been described as cognitive control, where the brain develops alternative neural pathways to maintain cognitive performance in the face of brain injury or disease (Hillary, 2008). The potential mechanisms underlying the cognitive changes observed in the hypertension group may be related to the adverse effects of hypertension on cerebral blood flow regulation and the blood–brain barrier (Iadecola, 2013). Additionally, chronic hypertension is associated with white matter lesions and cerebral microbleeds, which can disrupt neural connections and lead to cognitive decline (Debette and Markus, 2010). The NCT revealed no statistically significant differences, indicating that the cognitive impairments caused by hypertension may be in their early stages and that the differences have not yet reached significance.

In the diabetes group, patients showed reduced centrality in repetition and naming abilities. Our findings are consistent with some previous studies, such as the study by Biessels et al. (2006), which demonstrated that diabetic patients have an increased risk of cognitive dysfunction, particularly in memory, attention, and executive function domains. Another study by Cukierman et al. (2005) reported that diabetes is associated with a moderate decline in cognitive function, with a greater decline for individuals with poor glycemic control. Other confounding factors, such as glycemic control, duration of diabetes, or the presence of diabetes complications, may also influence the relationship between diabetes and cognitive function (Umegaki, 2014). The potential mechanisms underlying the cognitive changes observed in diabetic patients may be related to the adverse effects of hyperglycemia on the cerebrovascular system and neuronal function (Biessels et al., 2008; Grabenhenrich and Roll, 2014). Additionally, insulin resistance and chronic inflammation, which are common in diabetic patients, are associated with the development of cognitive impairments (Brownlee, 2001; Yaffe et al., 2004). The NCT revealed no statistically significant differences, suggesting that cognitive impairments may be in their early stages.

In the cerebrovascular disease group, patients exhibited weaker centrality in memory and spatial orientation. Moreover, the cognitive network structure became sparser, and the connections within cognitive domains were weaker. The NCT revealed significant differences in the overall strength of the intergroup network connections, indicating that cerebrovascular disease, a more advanced disease state, may have a more substantial impact on cognitive function than hypertension and diabetes. Cerebrovascular disease may disrupt the integrity of brain functional networks. Our findings are consistent with previous studies, showing that cerebrovascular disease is associated with a decline in cognitive abilities, particularly in memory and spatial orientation domains. A study by Jokinen et al. (2006) found that memory and executive functions were significantly impaired in patients with subcortical ischemic vascular disease. Similarly, a meta-analysis by Debette et al. (2011) reported that cerebrovascular disease is associated with a decline in cognitive abilities and an increased risk of dementia. The potential mechanisms underlying these observations may involve white matter lesions and cerebral small vessel disease, which are common in cerebrovascular disease patients and are associated with a decline in cognitive abilities (Pantoni, 2010; Prins and Scheltens, 2015). Additionally, impaired cerebral blood flow and the presence of cerebral microbleeds may contribute to the cognitive changes observed in cerebrovascular disease patients (Iadecola, 2013). Reduced cerebral blood flow could result in an insufficient oxygen and nutrient supply to the brain, ultimately leading to neuronal dysfunction and cognitive impairments (Iadecola, 2010).

In the coronary heart disease group, patients exhibited weaker centrality in memory, repetition, executive function, recall, attention, and calculation. In addition, the cognitive network structure became sparser, and connections within each cognitive domain were further weakened. The NCT revealed significant differences in the overall network structure and overall connection strength. Coronary heart disease may have a more substantial impact on cognitive function. Our findings are consistent with previous research, showing a relationship between coronary heart disease and cognitive decline in multiple domains. A study by Kure et al. (2016) found that coronary heart disease patients exhibited impairments in memory, attention, and executive function. Kresge et al. (2018) found that in subclinical cardiac disease, patients had poorer visual-spatial immediate recall, visual-spatial delayed recall, and verbal delayed recall. Similarly, a meta-analysis by Yang et al. (2020) reported that coronary heart disease is associated with a decline in cognitive abilities and an increased risk of dementia. The changes in centrality and sparse cognitive network structure observed in our study may be attributed to several underlying mechanisms. One possibility is that coronary heart disease can lead to chronic cerebral hypoperfusion, which in turn may result in neuronal dysfunction and cognitive impairments (Gorelick et al., 2011; Abete et al., 2014). Additionally, microvascular dysfunction and endothelial dysfunction, which are common in coronary heart disease, are associated with cognitive decline (Corona et al., 2012; Shabir et al., 2018).

## 5. Limitations

The current study has several limitations that should be considered when interpreting the results. First, given that this is an exploratory study using cross-sectional data, we could only identify possible associations between the four diseases and cognitive function, but causal inferences cannot be made. Second, the study estimated the networks based on group-level data. Inferring individual patients’ neurocognitive function from group-level data may be problematic, as the average situation of a population might not necessarily be relevant to individual patients. Future network studies could utilize longitudinal data to investigate individual differences and population patterns in neurocognitive function. Another advantage of using longitudinal data is the opportunity to statistically model the temporal dynamics of neurocognitive function, which may help elucidate patterns of cognitive impairment. Third, although we corrected for co-occurrence between diseases and the influence of factors such as gender, age, and education, there may still be other potential confounding factors that have not been accounted for in our analysis. Finally, we studied only four common cardiovascular-related chronic diseases of elderly individuals: hypertension, diabetes, cerebrovascular disease and coronary heart disease. Future studies could explore other potential risk factors and their relationship with cognitive function to gain a more comprehensive understanding of the influencing factors and protective mechanisms for cognitive function in the elderly population.

## 6. Conclusion

In summary, our exploratory analysis identified potential associations between four diseases and impairments in different cognitive domains. Our findings suggest that when screening for cognitive function in specific populations, greater attention should be given to the scores in particular cognitive domains to improve the accuracy and sensitivity of the screening. This may also provide useful insights for developing personalized cognitive intervention strategies for patients with cardiovascular diseases. Future research should further explore the potential mechanisms and intervention measures to prevent or mitigate cognitive decline in this population.

## Data availability statement

The datasets presented in this study can be found in online repositories. The names of the repository/repositories and accession number(s) can be found below: https://osf.io/vh825/?view_only=e18be553d28047788e5dfbc9303a2817.

## Ethics statement

The study protocol was approved by the Human Ethics Committee of China Medical University. The participants provided their written informed consent to participate in this study.

## Author contributions

YW: conceptualization, methodology, software, investigation, and writing—original draft. HZ: writing—review and editing, validation, and formal analysis. LL: writing—review and editing. ZL and JW: investigation. YaZ: formal analysis. YX: data curation and project administration. YiZ: project administration. YT: writing—review and editing, supervision, project administration, and funding acquisition. All authors contributed to the article and approved the submitted version.

## Footnotes

1
https://osf.io/vh825/?view_only=e18be553d28047788e5dfbc9303a2817
